# Legal and economic perspectives on fair and equitable benefit sharing in the Nagoya Protocol

**DOI:** 10.1111/cobi.14410

**Published:** 2024-10-22

**Authors:** Tae Jung Park, Sung‐Pil Park

**Affiliations:** ^1^ Graduate School of Future Strategy KAIST Daejeon Republic of Korea

**Keywords:** benefit sharing, development, economics, Indigenous people, intellectual property protection, law, Nagoya Protocol, desarrollo, economía, ley, protección de la propiedad intelectual, Protocolo de Nagoya, pueblos indígenas, reparto de beneficios

## Abstract

Adopted in 2010 as a supplementary agreement to the 1992 Convention on Biological Diversity, the Nagoya Protocol (NP) mandates the fair and equitable sharing of benefits arising from the use of genetic resources provided by Indigenous peoples. Member states must newly enact or amend domestic laws to align with the NP. Consequently, many countries are currently implementing legislative, administrative, and policy measures to ensure fair benefit sharing from the use of Indigenous genetic resources. We examined the inclusion of intellectual property (IP) protection in the sharing of benefits from research and development that utilizes Indigenous genetic resources. The NP does not specify guidelines for IP‐related benefit sharing, leaving each member state to establish its own rules. We used an economics‐based approach to explore the optimal scope and duration of IP protection for maximizing stakeholder interests, including those of Indigenous peoples, at the national level. The optimal duration of IP protection was when the marginal social cost and benefit of IP protection were equal. When this point occurred varied depending on various factors, such as the type of genetic resources in the country, existence of alternatives, number of users, and competing actors. The optimal scope of IP protection was when the social benefit of investment in fundamental research equaled the social benefit of application development. Likewise, this point of implementation also varied based on various factors, such as the type, uniqueness, potential for further discovery, and diversity of providers in the country.

## Abstract (es)

Perspectivas legales y económicas del reparto justo y equitativo de beneficios en el Protocolo de Nagoya

**Resumen:**

Adoptado en 2010 como un acuerdo complementario del Convenio sobre la Diversidad Biológica de 1992, el Protocolo de Nagoya establece el reparto justo y equitativo de los beneficios derivados del uso de los recursos genéticos proporcionados por los pueblos indígenas. Los Estados miembros deben promulgar o modificar su legislación nacional para adaptarla al Protocolo de Nagoya. En consecuencia, muchos países están aplicando actualmente medidas legislativas, administrativas y políticas para garantizar una participación justa en los beneficios derivados del uso de los recursos genéticos indígenas. Examinamos la inclusión de la protección de la propiedad intelectual (PI) en la participación en los beneficios derivados de la investigación y el desarrollo que utilizan recursos genéticos indígenas. El Protocolo de Nagoya no especifica directrices para la participación en los beneficios relacionados con la PI, dejando que cada Estado miembro establezca sus propias normas. Usamos un enfoque económico para estudiar el alcance y la duración óptimos de la protección de la propiedad intelectual con el fin de maximizar los intereses de los actores, incluidos los de los pueblos indígenas, a escala nacional. La duración óptima de la protección de la PI ocurría cuando el costo social marginal y el beneficio de la protección de la PI eran iguales. El momento en que se produce este punto varía en función de diversos factores, como el tipo de recursos genéticos del país, la existencia de alternativas, el número de usuarios y los actores que compiten entre sí. El alcance óptimo de la protección de la PI ocurría cuando el beneficio social de la inversión en investigación fundamental igualaba al beneficio social del desarrollo de aplicaciones. Asimismo, este punto de aplicación también varió en función de diversos factores, como el tipo, la singularidad, el potencial de nuevos descubrimientos y la diversidad de proveedores en el país.

## INTRODUCTION

The 1992 Earth Summit in Rio de Janeiro established Indigenous peoples as a “major group” under the Rio Conventions, which comprised the Convention on Biological Diversity (CBD), UN Framework Convention on Climate Change, and UN Convention to Combat Desertification. The UN Environment Programme identifies 9 major groups that it works with: farmers, women, the scientific and technological community, children and youth, Indigenous peoples and their communities, workers and trade unions, business and industry, nongovernmental organizations, and local authorities. The CBD specifically recognizes the significance of Indigenous peoples’ knowledge of and dependence on biological resources. The CBD preamble recognizes

the close and traditional dependence of many Indigenous and local communities embodying traditional lifestyles on biological resources…and the desirability of sharing equitably benefits arising from the use of traditional knowledge, innovations, and practices relevant to the conservation of biological diversity and the sustainable use of its components… (CBD, 2007a).

In particular, article 8(j) of the CBD stipulates the equitable sharing of benefits arising from the use of knowledge, innovations, and practices of local and Indigenous communities, as follows:

The member states shall, as far as possible and as appropriate, subject to its national legislation, respect, preserve and maintain knowledge, innovations and practices of indigenous and local communities embodying traditional lifestyles relevant for the conservation and sustainable use of biological diversity and promote their wider application with the approval and involvement of the holders of such knowledge, innovation and practices and encourage the equitable sharing of the benefits arising from the utilization of such knowledge, innovations and practices (CBD, 2007b).

However, because the CBD classifies biotechnological and industrial outputs as private property, some suggest that it potentially excludes Indigenous peoples from the commercialization process (McGonigle, 2016). In response to such criticisms of inadequate protection of Indigenous peoples’ commercial interests (Biber‐Klemm & Berglas, 2006), member states adopted the Nagoya Protocol (NP) in 2010. The NP aims to ensure the fair and equitable sharing of benefits arising from genetic resource utilization. Although it does not explicitly specify the expected socioeconomic outcomes, successful benefit sharing would presumably promote the socioeconomic development of Indigenous territories (Drahos & Frankel, 2012). Estimates suggest that allocating 10% of global profits from traditional knowledge (about US$500 billion) to Indigenous peoples could meet their basic needs (Teran, 2016). Ultimately, the economic benefits of intellectual property (IP) rights protection could also enhance the quality of life of Indigenous populations.

Many member states that provide or use genetic resources are enacting laws or revising existing ones to comply with NP provisions (Kamay, 2022). Such countries include Vietnam, Brazil, Ecuador, Peru, South Africa, Costa Rica, and Malaysia (Kamau, 2022). These laws will inevitably affect the daily lives of Indigenous peoples. Against this background, IP‐related benefit sharing has become increasingly important given the prevalent use of genetic resources in research and development (R&D), often resulting in the establishment of IP rights and subsequent benefit sharing through IP rights protection (Ruckstuhl et al., 2023). The NP lacks specific guidelines for IP‐related benefit sharing, delegating rule establishment to individual member states.

Focusing on practice and policy, we addressed the following question: What is the optimal duration or scope of IP protection that will maximize stakeholder interests, including those of Indigenous peoples, at national levels? We used an economics‐based theoretical framework to answer this question. Through our analysis, we sought to inform member states’ considerations of optimal levels when implementing the NP, which could then be incorporated into contracts between genetic resource providers and users to facilitate compliance with the NP.

## IP‐RELATED BENEFIT SHARING FOR INDIGENOUS PEOPLES

The CBD has 3 main objectives: conservation of biological diversity, sustainable use of its components, and fair and equitable benefit sharing that arises from the use of genetic resources. The third objective was established by the NP, which was adopted in 2010 by the Conference of the Parties (COP) and ratified by 51 member states in Pyeongchang in 2014 at COP 12. Article 5 (fair and equitable sharing of benefits), paragraph 2 of the NP requires member states to take legislative, administrative, or policy measures to ensure that the benefits arising from the use of genetic resources held by Indigenous peoples are fairly and equitably shared with those people. Article 5, paragraph 5 mandates the fair and equitable sharing of benefits arising from the use of traditional knowledge (CBD 2011, article 5). In addition, the NP stipulates that fair and equitable benefit sharing is to be achieved through mutually agreed terms (MATs), which are private agreements negotiated between Indigenous peoples and users who seek to utilize genetic resources or traditional knowledge (CBD 2011, article 5). In short, member states are required to establish legislative and administrative procedures for benefit sharing, and, based on those procedures, entities, such as businesses or research institutions, must enter into MATs with Indigenous peoples for the use of genetic resources or traditional knowledge associated with them.

As detailed in the NP's annexes, MATs outline ways to share monetary and nonmonetary benefits between the users and providers of genetic resources or traditional knowledge. Monetary benefits include royalties, licensing fees for commercialization, research funding, and benefits from the joint ownership of acquired IP rights. Nonmonetary benefits include the sharing of research and development results, participation in product development, and joint ownership of related IP rights (CBD 2011, annex).

The IP rights‐related provisions are critical in MAT negotiations between the providers (i.e., Indigenous peoples) and users of genetic resources. The CBD defines genetic resources as “genetic material of actual or potential value” and genetic material as “any material of plant, animal, microbial, or other origin containing functional units of heredity” (CBD, 1992). Thus, genetic resources comprise materials from any nonhuman living organism containing genes or derived biochemical compounds that are potentially beneficial for various purposes. Although genetic resources themselves are not creations and thus not subject to IP protections, various industries will use these resources in their research and development, generating results that are potentially eligible for IP protection. Likewise, traditional knowledge related to genetic resources might not initially be eligible for IP protection, but information resulting from research and development based on such knowledge can be eligible.

Therefore, the use of genetic resources and traditional knowledge could result in the establishment of various IP rights, such as patents, copyrights, trademarks, and trade secrets. Patents are commonly used to protect technology derived from genetic resources, especially in biochemical research, where unexpected inventions may emerge. Matters such as joint patent ownership, benefit distribution, and patent duration and scope are often addressed in MATs. Additionally, MATs can include provisions related to other IP forms, such as copyrights. Copyrights can apply to advanced characterization data from genetic resources or systematically organized traditional knowledge databases. Trademarks can distinguish resources, compositions, processes, or outcomes related to using genetic resources and traditional knowledge. Trade secrets may cover inventions derived from genetic resources that do not meet patentability criteria but remain commercially valuable.

In summary, the NP aims to ensure fair and equitable benefit sharing between Indigenous peoples and the users of genetic resources and traditional knowledge. Member states are required to take measures to facilitate such benefit sharing, forming a basis for MAT negotiations between users and Indigenous peoples. An MAT typically includes provisions for IP protection, especially regarding patents. The NP, however, lacks specific guidelines for IP‐related provisions in laws or MATs. Although recommending procedures, such as obtaining Indigenous peoples’ informed consent and implementing benefit‐sharing mechanisms, the NP only notes that such measures should be “appropriate,” giving member states leeway to determine the entire ruleset. Member states must formulate policy proposals related to IP provisions, but there is no specific guidance on IP provisions for MATs. Although article 19 of the NP encourages countries to develop contractual agreements for MATs and urges Indigenous people to utilize them, it provides no specific guidance on IP provisions. Consequently, member states must exercise judgment regarding IP‐related benefit‐sharing rules. Similarly, genetic resource users and providers both have to self‐judge IP‐related benefit sharing through MATs.

## OPTIMUM LEVEL OF BENEFITS PROTECTION FOR INDIGENOUS PEOPLE

In the absence of specific NP guidance on IP‐related benefits, we explored the optimal scope and duration of IP that can maximize the interests of stakeholders, including genetic resource users, providers, and governments. To this end, we used a marginal analysis‐based economics approach.

There is no universal law regulating Indigenous people's benefits. The law and development literature suggests that countries should establish their own laws based on local cultures and contexts (Prado & Trebilcock, 2021). In this process, they should consider elements such as IP types and context, Indigenous customary laws, preexisting access to benefit‐sharing community protocols, institutional quality, and enforcement capacity. Legal transplantation (Husa, 2018) from a developed country would be inefficient for determining appropriate IP provisions. Rather, member states should devise their own IP‐related benefit‐sharing rules, as opposed to copying laws from developed countries or those with a history of protecting Indigenous people.

**FIGURE 1:**
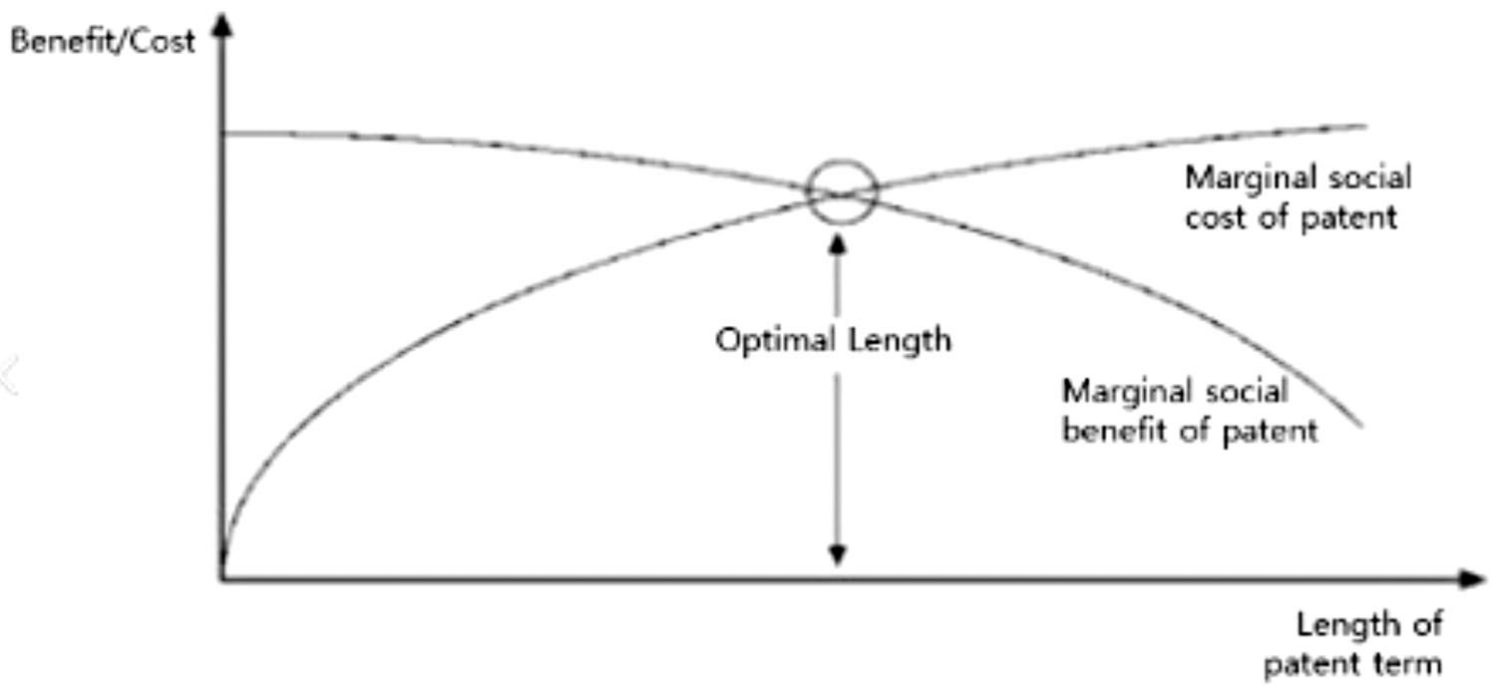
Optimal duration of protection of intellectual property (e.g., patents).

The appropriate duration for IP protection is determined by balancing social cost and benefit (Park et al., 2019). Social costs may include higher product prices due to IP protection and efforts to find alternative genetic resources or traditional knowledge. The social benefits of IP protection can include providing economic incentives for ongoing innovation by prohibiting free riding (Park et al., 2019). Theoretically, the optimal IP protection duration corresponds to when the marginal social benefit equals the marginal social cost (Park et al., 2019). Marginal social benefit refers to the benefit of extending IP protection by one unit (e.g., 1 year), and marginal social cost is the cost of such an extension. Extending the duration of IP protection could increase social costs at an accelerating rate, resulting in a marginal social cost curve that slopes upward. Although a longer duration of IP protection also enhances social benefit by encouraging research and development, the rate of this increase diminishes, producing a downward‐sloping marginal social benefit curve. Thus, the optimal IP protection duration corresponds to the intersection of the curves of marginal social benefit and cost (Figure 1; Park et al., 2019).

Various elements influence these costs and benefits, including genetic resource types, the existence of alternatives, the number of users, and competing actors. These factors vary significantly across different regions. Some genetic resources and associated traditional knowledge may be unique, whereas others are easily replaceable. The range and number of users and providers also differ by region. These variables significantly affect protection costs and benefits, resulting in optimal durations that are specific to each country and region.

The scope of IP protection varies by region and is especially challenging when new inventions stem from prior discoveries. Although pioneering discoveries may hold vast commercial potential, they initially lack commercial value until further research transforms them into marketable inventions. Countries face the decision of whether to extend IP protection to these subsequent inventions. Theoretically, protection should expand if the social value of investment in fundamental research outweighs that in application development, and it should narrow if the opposite is the case (Cooter & Ulen, 2016). In short, if the social benefits of the first discovery exceed those arising from the development of such a discovery, then the protection of the pioneering discovery should be broadened, and vice versa.

The market potential and subsequent invention allowance for genetic resources or traditional knowledge also vary based on uniqueness and other characteristics. In regions inhabited by Indigenous peoples, broader protections may be warranted given the higher social benefits of pioneering discoveries. By contrast, other regions might warrant narrower protections when there is less social benefit from initial discoveries. Therefore, the scope of protection for genetic resources is affected by their type and uniqueness, their potential for further discovery, and the diversity of providers, leading to significant regional variation.

Although pinpointing the optimal IP level is challenging, member states need to gather data and aim to identify it through collaboration among research institutes, academia, and industry. Even if the optimal level is elusive, it is necessary to estimate it and have this estimate reflected in domestic laws. The NP does not prescribe specific domestic laws but allows countries discretion in this area. Thus, countries should consider integrating the concept of an optimal IP level in their domestic legislation considering scope and duration. Moreover, the optimal patent scope and duration should be mirrored in material transfer agreements. Article 19 of the NP requires countries to develop contractual agreements for MATs and promote their use. Fair benefit sharing can be achieved through material transfer agreements that incorporate optimal IP provisions, aligning with the interests of both the users and providers of genetic resources.

## CONCLUSIONS

With the adoption of the NP, member states are enacting laws to ensure fair and equitable benefit sharing following the use of genetic resources and associated traditional knowledge owned by Indigenous peoples. This process aims to achieve fair sharing through MATs, which are private contracts between genetic resource users and Indigenous peoples. Mutually agreed terms outline various monetary and nonmonetary benefits, including IP rights provisions.

The scope and duration of protection for IP arising from research and development based on Indigenous peoples’ genetic resources need to be considered. For patents, the optimal scope and duration vary based on factors, such as country, region, the nature of the genetic resources, related industries, and the number of users and providers. Although it is difficult to quantify specific durations and scopes, our proposed theoretical framework can be used to enact national laws and negotiate MATs for fair benefit sharing.
